# Comparison of the Evaluation of Combination of Ultrasonography of the Reproductive Tract With Hormone Administration on Dairy Cow Fertility

**DOI:** 10.3389/fvets.2022.840724

**Published:** 2022-03-15

**Authors:** Bingke Wang, Jinbang Xiao, Yongjie Ma, Chuxi Gao, Hanbing Li, Yonghong Jia, Yaping Jin, Pengfei Lin

**Affiliations:** ^1^College of Veterinary Medicine, Northwest A&F University, Yangling, China; ^2^Key Laboratory of Animal Biotechnology, Ministry of Agriculture and Rural Affairs, Northwest A&F University, Yangling, China; ^3^Yangling Nongfu Agriculture and Animal Husbandry Technology Co., Ltd., Yangling, China; ^4^Animal Husbandry Industry Test and Demonstration Center of Shaanxi Province, Xianyang, China

**Keywords:** reproduction program, ultrasonography, endometritis, hormone, dairy cow

## Abstract

Further optimization of reproduction management programs in dairy cows is a contemporary research topic. In this context, our study aimed to compare a hormone program, named “uterus-ovary monitoring and classified use of hormone program” (M+C), with the Pre-OvSynch program. The M+C was based on regular application of B-mode ultrasonography during a voluntary waiting period to monitor the uterus and ovaries, while using various treatments under different conditions. Results of the 30–33-day and 60-day pregnancy/artificial insemination after the first AI of M+C were significantly better than the Pre-OvSynch (*p* < 0.05). The pregnancy rates within 180 days in milk after M+C was significantly higher than that after Pre-OvSynch (*p* < 0.05). The total number of inseminations used for M+C was significantly lower than that for Pre-OvSynch (*p* < 0.01). The number of open days was fewer after M+C than after the Pre–OvSynch throughout the experimental period with highly significant differences (*p* < 0.01). In summary, the use of M+C enhances reproductive benefits and reduces the need for hormone drugs among cows.

## Introduction

Fertility in dairy cows is a major factor affecting the stable growth of core cattle herds and a sustained increase in milk production. It also has direct effects on the economic benefits of dairy farms (1). Improving cow fertility remains a highly researched issue worldwide as fertility is closely related to many factors, including nutrition (2), genetics (3), environment (4), disease (5), and stress (6), as well as to the implementation and optimization of the relevant reproduction programs.

Since its development, the synchronization of ovulation (OvSynch) and timed artificial insemination (TAI) program (OvSynch+TAI) has significantly reduced the time of the waiting period and the workload of reproductive workers. The Pre-OvSynch, Double-OvSynch, and ReSynch programs (7), all of which are based on the OvSynch program, have been optimized for large-scale batch production management. Nevertheless, some OvSynch+TAI programs have demonstrated practical problems such as long handling time and the number of handlings, usage of hormones, high drug costs, and lack of screening for uterine and ovarian subclinical diseases before beginning the OvSynch protocol.

Endometritis, mainly due to bacterial infection, and ovarian dysfunction, caused by endocrine disorders, is the main reason for low postpartum reproductive function and delayed recovery (7, 8). These conditions severely affect the outcome of OvSynch+TAI and reduce reproductive capability. In addition to synchronization programs, hormone drugs can also be used to treat reproductive disorders (9); for example, gonadotropin-releasing hormone (GnRH) is used to treat ovarian dysfunction caused by ovarian cysts and postpartum anestrus (10). Moreover, prostaglandin F2α (PGF2α), which initiates luteolysis, can be used to treat corpus luteum cysts, and promotes tissue debris excretion and inflammatory secretion from the uterus, thus preventing and resolving endometritis (11). Nevertheless, a recent meta-analysis on the effect of PGF2α in cows undergoing endometritis treatment reported that PGF2α cannot be recommended to treat cows to improve their reproductive performance (12).

Hormone drugs should be used with caution in clinical practice. The causes of reproductive disorders are complicated, and hormone treatment alone does not show optimal curative results. Moreover, the wide use of hormones also results in economic waste. Although the side effects of hormone drugs have long been taken seriously in clinical medicine (13), relevant research in cattle remains insufficient. In dairy farms, the use of routine monitoring and disease detection has been widely applied for effective disease detection and early intervention. Ultrasonography provides unique advantages for examining organ status. With further development, ultrasonography could be accurately and conveniently used to monitor the status of both the uterus (14) and the ovaries (15), making it a practical and effective method for diagnosing endometritis and ovarian diseases (16, 17). Thus, the development and promotion of ultrasonography for disease monitoring demonstrate high potential (18).

Therefore, in this study, in order to effectively diagnose and treat endometritis, reduce the use of hormones, improve the reaction of dairy cows to the OvSynch program before AI, and optimize reproductive efficiency comprehensively, we develop and evaluate a precise hormone program, named the “uterus-ovary monitoring and classified use of hormone program” (M+C), based on regular application of B-mode ultrasonography during a voluntary waiting period to monitor the uterus and ovaries.

## Materials and Methods

### Cows and Dairy Farm

This experiment was conducted at a commercial dairy farm in Northwest of China, from June to August 2020. At the time of the experiment, this farm had 3,826 Holstein cows, of which 1,708 were lactating; their average parity number was 2.65, and average annual milk yield was 9,134 kg. All cows were fed a TMR three times a day and managed using the herd management system “Aladdin” (Beijing HemuXingbang Network Technology Co., LTD., China). The hardware facilities, diet formulation, staff, and herd structure remained consistent during the experimental period. Within 60 days after delivery in this farm during the whole experiment period, incidence of lameness, ketosis, placental retention, metritis, and milk fever were 5.1, 12.3, 1.9, 2.4, and 4.2%, respectively. After calving, postpartum cows with different parity were randomly divided into two groups, M+C group and Pre-OvSynch group, respectively, and the voluntary waiting period was 60 days for both. Finally, the cows that received the complete M+C (*n* = 262) or Pre-OvSynch (*n* = 264) program were included in subsequent statistical analysis. All animal handling procedures were approved by the Committee for the Ethics on Animal Care and Experiments at Northwest A&F University.

### M+C and Pre-OvSynch Program

**Figure 1:**
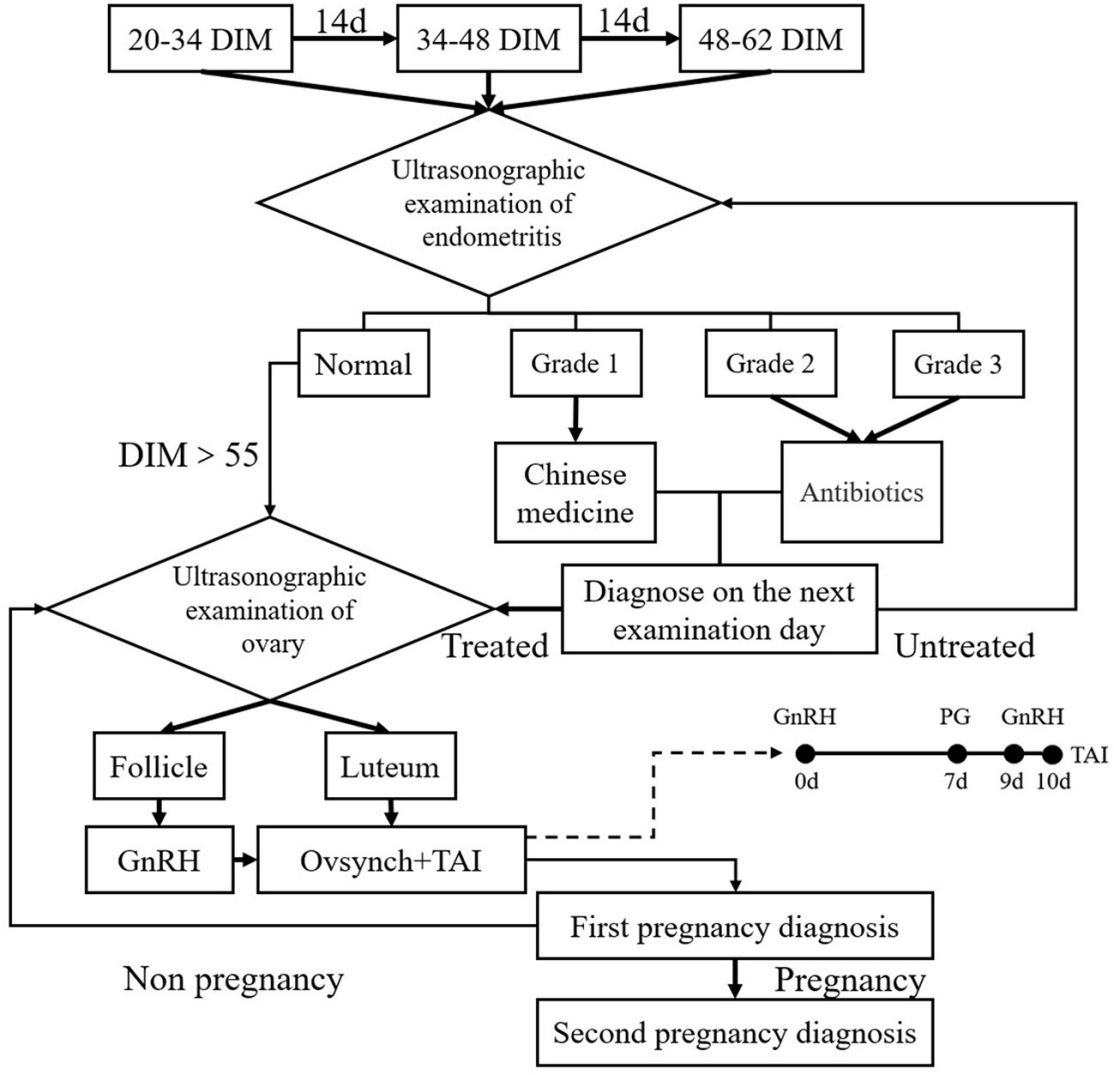
Schematic diagram of the M+C program. Each cow underwent three regular reproductive organ inspections before insemination. The first inspection was performed at 20–34 days in milk (DIM). B-mode ultrasonography was used to examine the uterus and ovary transrectally and to take appropriate measurements under different conditions. After 14 and 28 days, the same examination and treatment were performed again. At 20–34, 34–48, and 48–62 DIM, the images for cow uterus size and abnormal fluid in uterus cavity were classified into four grades: normal and endometritis level I to III; Chinese medicine was used to treat cows with endometritis, and antibiotics were used to treat cows with endometritis level II or III. If a cow was diagnosed with endometritis during examination and treated, we waited 14 days until the next examination for diagnosis. In all cows with endometritis, TAI was performed only after endometritis was resolved. Then, if DIM was >55 at this time, different treatment protocols were adopted for cows without abnormalities depending on their ovary phase. If the corpus luteum was mature with a still developing ovarian follicle in the cow ovary (i.e., the luteal phase), then we applied OvSynch+TAI; if the cow ovary had a mature ovarian follicle (i.e., the follicular phase), GnRH was used first, followed by OvSynch+TAI after 7 days.

In the M+C program, each cow underwent three regular reproductive organ inspections using B-mode ultrasonography (British BCF, model Easi-scan3) by an experienced technician before artificial insemination (Figure 1). The three examinations were performed at 20–34, 34–48, and 48–62 days in milk (DIM). Subsequently, different treatment protocols were adopted for cows depending on the ovarian cycle phase as examined by B-mode ultrasonography. If there was a mature corpus luteum in the ovary, a OvSynch+TAI protocol was chosen; GnRH (100 μg/stem; Ningbo Sansheng Pharmaceutical, China) was injected first (Day 0), followed by an injection of PGF2α (25 mg/pc, Ningbo Sansheng Pharmaceutical, China) on Day 7, and another GnRH injection on Day 9. If there was a mature follicle in the ovary (follicular phase), a pre-injection of GnRH was given, followed after 7 days by a complete OvSynch+TAI protocol as above. The typical ultrasound imaging findings of the mature corpus luteum were given as uniform gray image of regular boundary with 2–3 cm long and 1.5–2.5 cm wide. Mature follicles range from 1.5 to 2.0 cm in diameter (long axis).

In the Pre-OvSynch program, no ultrasonography took place but cows were treated with 25 mg of PGF2α at 14–21 DIM, followed by two 25-mg treatments of PGF2α at 14-day intervals, followed after 11 days by a complete OvSynch+TAI protocol.

Cows were scheduled to receive TAI ~16 h after the last GnRH treatment of the M+C and Pre-OvSynch protocols. The artificial insemination personnel were randomly assigned to three artificial insemination technicians on the dairy farm. The first pregnancy diagnosis through B-mode ultrasonography was performed by experienced veterinarians 30–33 days after TAI to observe signs of pregnancy. The second pregnancy diagnosis was performed 60 days after TAI to determine pregnancy loss. Cows that were not pregnant repeatedly underwent ultrasonographic examination of the ovary in M+C (Figure 1), or perform the OvSynch+TAI program in the Pre-OvSynch group.

### B-Mode Ultrasonography for Diagnosis of Endometritis Followed by Classification and Treatment

In this study, uterus size and abnormal uterine fluids at 20–34, 34–48, and 48–62 DIM were examined using B-mode ultrasonography (19, 20), and then classified into four grades based on endometritis occurrence and severity:

1. No endometritis (Normal, Grade 0): No abnormal findings were noted in the uterus (no fluid or echo-free); the cervical diameter was <7.5 cm, and no abnormalities were noted on clinical examination.

2. Endometritis grade I: A small amount of abnormal fluid (discontinuous hyperechogenic, thin <1 mm) was detected in the uterus.

3. Endometritis grade II: A considerable amount of abnormal fluid (continuous hyperechogenic, thin ≥1 mm) was detected in the uterus.

4. Endometritis grade III: A considerable amount of abnormal fluid was detected in the uterus, accompanied by purulent fluid accumulation with some fluctuation (large amount of storage material that looked like a snowstorm).

Clinical examination of the cow tail, perineum, and vulva for purulent secretion in the uterus was also performed. If uterine purulent secretion was detected, the cows were directly classified with endometritis grade III.

For cows with endometritis grade I, Gongyankang (commercial Chinese medicine pills, with active ingredients including *Epimedium, Actinolite, Angelica sinensis, Cyperus rotundus, Leonurus japonicus*, and *Sophora flavescens*; 10 capsules/bottle; Jilida, Jilin, China) were administered by uterine infusion. Cows with endometritis grade II or III were given antibiotics (21) (10% oxytetracycline uterine injectant; Eastern Along Pharmaceutical Co., LTD; China). If cows were diagnosed with endometritis, they would receive a course of treatment, and then receive B-mode ultrasonography diagnose on the next examination (14 days later) to determine therapeutic effect. Endometritis was considered completely cured when B-mode ultrasonography and clinical examination were at normal grades. For all cows with endometritis, TAI was only performed when endometritis was cured. The number of cows diagnosed as returning to grade 0 before AI expressed as a percentage of the total number of cows diagnosed with endometritis.

### Statistical Analysis

All data are presented as the means ± standard error of the mean (SEM) and analyzed using SPSS (version 24.0). Logistic regression was used to analyze the pregnancy rates at 30–33 and 60 days after the first AI. Survival analysis with right censoring was used to analyze pregnancy rates within 180 DIM. The chi-square test was used to analyze the incidence and rate of endometritis. One-way analysis of variance was used to analyze the number of frozen semen straws, the cost of drugs, hormones, and frozen semen. Differences were considered significant when *p* < 0.05.

## Results

### Effects of M+C and Pre–OvSynch Program on P/AI After First AI of Post-partum Cows

As shown in Table 1, no significant difference in parity was observed between the two programs (*p* > 0.05). The 30–33 and 60-day P/AI of M+C were significantly higher than those of Pre-OvSynch (*p* < 0.05). In addition, the pregnancy loss was not significantly different between the two programs (*p* > 0.05). The above results indicated that M+C might significantly improve P/AI after the first AI of postpartum cows.

**Table 1:** The parity, 30–33-day P/AI, 60-day P/AI after first AI, and pregnancy loss of postpartum cows treated with the M+C and Pre-OvSynch program.

**Item (%, *n*/*n*)**	**M+C**	**Pre-OvSynch**	***p*-value**
Parity (*n*)	2.47 ± 1.12	2.49 ± 1.16	0.817
30–33-day P/AI (%, *n*/*n*)	52.3 (137/262)^a^	41.7 (110/264)^b^	0.015
60-day P/AI (%, *n*/*n*)	47.3 (124/262)^a^	36.7 (97/264)^b^	0.014
Pregnancy loss (%, *n*/*n*)	9.5 (13/137)	11.8 (13/110)	0.594

*The data of the M+C and Pre-OvSynch programs in the table include cows with and without endometritis. Different superscripts between columns indicate significant difference (p < 0.05)*.

### Pregnancy Rates Within 180 DIM, Number of Frozen Semen, and Cow Open Days With M+C and Pre–OvSynch Program

The pregnancy rates within 180 DIM after M+C was significantly higher than that after the Pre–OvSynch program (Table 2, *p* < 0.05). The total number of frozen semen used for M+C was significantly lower than that of Pre–OvSynch (Table 2, *p* < 0.05). Meanwhile, the number of open days was significantly lower in M+C than in Pre–OvSynch (Table 2, *p* < 0.05). These results suggest that the application of M+C could significantly reduce the number of frozen semen straws and the number of open days.

**Table 2:** The pregnancy rates within 180 DIM, number of frozen semen, and cow open days of the M+C and Pre–OvSynch program.

**Item**	**M+C**	**Pre-OvSynch**	***p*-value**
Pregnancy rates within 180 DIM (%, *n*/*n*)	92.4 (242/262)^a^	89.4 (236/264)^c^	0.002
Frozen semen straws (*n*)	1.95 ± 1.25^c^	2.32 ± 1.34^a^	0.002
Open days (days)	91.89 ± 34.43^c^	103.83 ± 37.89^a^	<0.001

*The data of the M+C and Pre-OvSynch programs in the table include cows with and without endometritis. Different superscripts between columns indicate significant difference (p < 0.05)*.

### Ultrasonography-Based Diagnosis of Endometritis and Ovaries in M+C Program

Ultrasonography for the diagnosis of endometritis in M+C was performed at 20–34, 34–48, and 48–62 days, respectively (Table 3). Approximately 15% of cows were diagnosed with endometritis of grade I at 20–34 days postpartum, whereas the diagnostic rates gradually declined at 34–48 (about 7%) and 48–62 days (about 3%), respectively. Meanwhile, the total diagnostic rates of endometritis in grades II and III at 20–34 days postpartum were 6.5% and 6.1%, respectively, and the diagnostic rates also gradually declined at 34–48 and 48–62 days.

**Table 3:** Ultrasonography-based diagnosis of endometritis in the M+C program.

**DIM (d)**	**Grade 0 (%)**	**Grade I (%)**	**Grade II (%)**	**Grade III (%)**
20–34	71.7	15.7	6.5	6.1
34–48	83.2	7.6	4.6	4.6
48–62	91.5	3.1	2.7	2.7

To evaluate the effect of treatment of cases with different grades of endometritis, the cure rate and 60-day P/AI after the first AI of cows were analyzed (Table 4). As the endometritis grade increased, the cure rate and 60-day P/AI after the first AI decreased; the cure rate for endometritis grade I was significantly higher than that for endometritis grade III (*p* < 0.05). However, no significant differences were detected in 60-day P/AI after the first AI among the three endometritis grades (*p* > 0.05).

**Table 4:** Cure rate and 60-day P/AI after the first AI of cow with endometritis in the M+C program.

**Grade**	**Number^1^**	**Cure rate^2^**	**60-day P/AI**
0 (Normal)	180	-	50.6%
I	48	75.0%^a^	45.8%
II	18	55.6%^bc^	38.9%
III	16	37.5%^c^	25.0%

1 *Number = The total number of cows with the first diagnosis at this grade on three inspection days*.

2 *Cure rate = Number of cows diagnosed as normal before AI/Total number of cows diagnosed with endometritis. Different superscripts between columns indicate significant difference, p < 0.05*.

The percentages of corpus luteum and ovarian follicles in the ovary at the first implementation of the OvSynch program in M+C were 82.4 and 17.6%, respectively, with those of 60-day P/AI after the first AI being 44.9 and 58.7%, respectively (Table 5, *p* > 0.05).

**Table 5:** Ultrasonography-based diagnosis of ovaries at the first implementation of the OvSynch program and 60-day P/AI after the first AI in M+C program.

**Ovarian cycle**	**Number of cows**	**Proportion**	**60-day P/AI**
Corpus luteum	216	82.4%	44.9%
Ovarian follicle	46	17.6%	58.7%

*The data of the M+C and Pre-OvSynch programs in the table include cows with and without endometritis*.

### Cost of Drugs, Hormone, and Frozen Semen in M+C vs. Pre–OvSynch in 180 Days

As shown in Table 6, in addition to the extra drug cost in M+C, the average cost of hormone and frozen semen was significantly lower in M+C than in Pre-OvSynch (*p* < 0.05).

**Table 6:** Cost of drugs, hormone, and frozen semen in M+C and Pre–OvSynch program during 180 DIM.

**Item**	**M+C**	**Pre-OvSynch**	***p*-value**
Cost of drugs (USD/CNY)	1.77 ± 2.19/12.13 ± 15.05	0.00 ± 0.00	<0.001
Cost of hormone (USD/CNY)	12.50 ± 6.75/85.73 ± 46.29^c^	25.24 ± 7.06/173.14 ± 48.45^a^	<0.001
Cost of frozen semen (USD/CNY)	9.95 ± 6.59/68.27 ± 45.18^c^	12.00 ± 7.22/82.30 ± 49.55^a^	0.001

*Different superscripts between columns indicate significant difference, p < 0.05*.

## Discussion

Increased pregnancy rate, reduced number of frozen semen straws used, and shortened number of open days are ideal attributes for dairy farms. At the same time, reducing the workload of reproductive workers in farms is also essential. Accordingly, the development and application of OvSynch+TAI has achieved remarkable results (22). Further optimization of synchronization programs has been a dynamic topic of research, mainly focusing on increasing hormonal management of reproduction (23), evaluating pregnancy diagnosis time (24), using novel drugs (25), enhancing estrus and hormone monitoring (26), and monitoring the ovary cycle (27). It is well-known that the OvSynch program provides the best results when it is started at day 5–9 of the estrous cycle, and the Pre-OvSynch program was developed for this purpose, but increasing hormone use. Nevertheless, increased hormone dosage and frequency may reduce personnel working enthusiasm and potentially lead to negative effects. For example, Pre-OvSynch may increase the proportion of male calves (28).

Funakura et al. (29) reported that using a portable ultrasound device to objectively evaluate the presence or absence of a functional corpus luteum could achieve a higher pregnancy rate than the conventional AI method based on estrus detection after administration of PGF alone in beef herds. Silva et al. (30) also concluded that assessing the corpus luteum functionality of Holstein cows using ultrasound at the first GnRH injection of Pre-OvSynch protocol could resolve the anovular condition before TAI and thus improve reproductive performance. In addition, using the synchronization program based on functionality of the corpus luteum as determined by ultrasonography may be a viable alternative to OvSynch, with an additional benefit of having lower costs (31). Similarly, in the current study, before performing the synchronization programs, we first examined the ovaries using B-mode ultrasonography. If a corpus luteum with a developing dominant follicle was noted on days 5–12 of the estrus cycle, the cow was considered to have high sensitivity to GnRH, and the OvSynch+TAI procedure was performed directly. If mature follicles were present, GnRH was used first to promote follicular expulsion and formation of a new corpus luteum, followed by the OvSynch program 7 days later to induce a new follicular cycle and ovulation. The results showed that compared with the Pre–OvSynch, M+C significantly increased the P/AI of cows, reduced the number of frozen semen straws required, shortened the number of open days, and reduced the usage of hormone drugs.

The incidence of endometritis, which affects the internal environment of the uterus and destroys normal ovary function, resulting in reduced postpartum fertility in cows, can reach up to 40% (32). Endometritis can be divided into clinical and subclinical types, with the former demonstrating typical clinical signs (33), while the latter usually requires endometrial cytology, biopsy, and leukocyte esterase testing to confirm the diagnosis (34), all of which are difficult to implement in dairy farms. Currently, endometritis is usually diagnosed after 21 DIM once in farms (16, 20); however, because ultrasonography inspection can be susceptible to the probe position and orientation and the uterine state of cows (35), it cannot be effectively used for diagnosing subclinical cases where no secretions are observed in the uterus (20). Hence, selecting multiple time points for checking can help improve sensitivity. PGF2α and intrauterine infusion of antibiotics are commonly used for endometritis treatment, but their effectiveness remains controversial (12, 36); however, the use of some herbal extracts has been investigated in the treatment of endometritis because they have antibacterial and anti-inflammatory characteristics, cause less irritability, and do not require milk to be discarded (12, 37). Based on the above reasons, in the current study, the M+C program referred and optimized the diagnosis method of endometritis used by Lenz et al. (19) and Kasimanickam et al. (17). The results showed a high incidence of endometritis at all grades in this trial within 20–34 days postpartum, with an overall diagnostic rate of 26.3%. The cure rate for endometritis grade I was significantly higher than that for endometritis grade II and III, and may be due to *L. japonicus* and *S. flavescens* as the main ingredients of Gongyankang have been reported to have a beneficial anti-inflammatory effect on endometritis (38, 39). However, our outcome for endometritis II and III treatment is still not ideal, and follow-up studies focusing on improving the treatment of severe endometritis are warranted. The M+C exhibits two advantages: first, endometritis cases were detected in a timely and effective manner through three regular examinations of the uterus before insemination. The effect of the treatment could be assessed on the next examination day, ensuring that the internal environment of the uterus is improved as much as possible before ovulation synchronization is performed. Second, cows categorized as having either mild or severe endometritis could be treated more specifically using different drugs, thus avoiding large-scale, blind use of hormone drugs, reducing drug costs, and achieving satisfactory results.

The measurement of economic benefits is an important part of evaluating the effectiveness of any program. In the evaluation of the optimized OvSynch+TAI program, the measurement of economic benefits usually requires a large amount of data and mathematical modeling, and based on preliminary analyses, compared with the Pre–OvSynch, M+C could reduce the amount of hormone drugs and frozen semen used; however, the costs of treatment drugs and B-mode ultrasonography were increased. In the future, we aim to continually improve and promote M+C and collect data to completely evaluate the cost of the different echographic examinations and the required time to make such examinations.

## Conclusions

The advantages of using M+C include effective endometritis detection, classification, and treatment and precise hormone administration according to the ovary cycle. These enhance reproductive benefits and reduce the need for hormone drugs in cows. Thus, M+C is convenient and feasible with value for promotion and application.

## Data Availability Statement

The original contributions presented in the study are included in the article/supplementary material, further inquiries can be directed to the corresponding author/s.

## Ethics Statement

The animal study was reviewed and approved by Committee for the Ethics on Animal Care and Experiments in Northwest A&F University (Approval ID: 2011ZX08008-002). Written informed consent was obtained from the owners for the participation of their animals in this study.

## Author Contributions

BW, YJin, and PL: conceptualization and resources. BW: methodology. JX and YM: software. CG, YJia, and HL: validation. JX: formal analysis. BW and JX: investigation. JX and PL: writing—original draft preparation. BW, JX, YM, CG, HL, YJia, YJin, and PL: writing—review and editing. YJin and PL: supervision. All authors contributed to the article and approved the submitted version.

## Funding

This research was funded by the Key Research and Development (R&D) program in Ningxia Hui Autonomous Region (2021BBF02037; 2018BBF33001) and the Science and Technology Innovation Project of Shaanxi Province.

## Conflict of Interest

BW was employed by Yangling Nongfu Agriculture and Animal Husbandry Technology Co., Ltd. The remaining authors declare that the research was conducted in the absence of any commercial or financial relationships that could be construed as a potential conflict of interest.

## Publisher's Note

All claims expressed in this article are solely those of the authors and do not necessarily represent those of their affiliated organizations, or those of the publisher, the editors and the reviewers. Any product that may be evaluated in this article, or claim that may be made by its manufacturer, is not guaranteed or endorsed by the publisher.
